# Mind over platter: pre-meal planning and the control of meal size in humans

**DOI:** 10.1038/ijo.2014.83

**Published:** 2014-07-25

**Authors:** J M Brunstrom

**Affiliations:** 1 Nutrition and Behaviour Laboratory, School of Experimental Psychology, University of Bristol, Bristol, UK

## Abstract

It is widely accepted that meal size is governed by psychological and physiological processes that generate fullness towards the end of a meal. However, observations of natural eating behaviour suggest that this preoccupation with within-meal events may be misplaced and that the role of immediate post-ingestive feedback (for example, gastric stretch) has been overstated. This review considers the proposition that the locus of control is more likely to be expressed in decisions about portion size, before a meal begins. Consistent with this idea, we have discovered that people are extremely adept at estimating the ‘expected satiety' and ‘expected satiation' of different foods. These expectations are learned over time and they are highly correlated with the number of calories that end up on our plate. Indeed, across a range of foods, the large variation in expected satiety/satiation may be a more important determinant of meal size than relatively subtle differences in palatability. Building on related advances, it would also appear that memory for portion size has an important role in generating satiety after a meal has been consumed. Together, these findings expose the importance of planning and episodic memory in the control of appetite and food intake in humans.

## Meal planning and the control of food intake

Portion-size matters—when offered larger amounts of food people reliably consume a larger meal. This is the case irrespective of the type of food that is offered^1,2^ and whether or not it is pre-packaged.^3,4^ As portion sizes have become larger,^5, 6, 7^ there is a concern that we are being coerced to overconsume and that volitional control is easily overridden by environmental influences. Estimates of area or volume are often prone to systematic error, especially when we rely too heavily on one particular dimension.^8^ This type of low-level perceptual bias may be responsible for the ‘portion-size effect.' Alternatively, large portions might increase social norms around what is considered typical or acceptable.^9,10^ These hypotheses are extremely important because they relate to phenomena that have a marked effect on food intake.^11^ However, to advance these ideas further, we need to know more about the specific process by which sensory and cognitive processing influence meal size in humans.

The prevailing view is that meal size can be understood by exploring psychological and physiological events that promote satiation (fullness) during and towards the end of a meal.^12, 13, 14^ Typically, participants are offered unlimited food and the dependent variable of interest is the amount eaten. However, by focusing on within-meal processes, there is a danger that we overlook meal planning as an essential component of dietary control.^15,16^ Several large-scale observational studies show that humans plan the amount of food that they are going to eat in advance of eating.^17, 18, 19^ This appears to be the case irrespective of the type of meal, (breakfast, lunch or dinner) and it reflects a general tendency either to ‘plate clean' or at least to be unsurprised by the amount remaining at the end of a meal.^20^ Meal planning has been observed under natural conditions in a restaurant^21^ and it is also evident in comprehensive and detailed qualitative analyses of consumers' interactions with food portions.^22^

This overwhelming tendency to plan a meal may reflect an underlying biological imperative.^15^ In early *Homo erectus*, the invention of cooking can be traced back to a decrease in gut size and an increase in brain size.^23^ Eating raw food is potentially unhealthy,^24^ and cooking eliminates pathogens and increases the energy that can be extracted from food.^25^ Planning serves an important functional role because it saves both time and energy. It enables us to predict the amount of food that is required before a meal is prepared, cooked and then served. Consistent with this idea, studies that remove the opportunity to plan and monitor food intake tend to generate aberrant overconsumption. For example, when people eat from a self-refilling soup bowl, meal size increases dramatically without a concomitant increase in self-reported fullness.^26^ Similarly, covert intra-gastric infusions of soup produce a blunted satiety response. Remarkably, this is partially restored when participants are told that they have been infused with soup.^27^ Thus, it would appear that the opportunity to know what and how much food has been consumed has a central role in satiety and the control of meal size.

## Meal planning and satiety

In several studies, Higgs *et al.*^28,29^ have shown that reminding people of a recent meal can decrease the amount that is consumed at a subsequent meal. This role for a ‘memory for recent eating' is consistent with emerging evidence that implicates hippocampal-dependent memory mechanisms in behavioural responses to food.^30^ In humans, the most dramatic example is found in patients with retrograde amnesia. After consuming a meal to fullness, an amnesic will report no memory for recent eating. In the absence of an ability to attribute visceral sensations to a particular ingestive event, hunger persists and hyperphagia is observed.^31, 32, 33^

The prospect that memory has this role invites the possibility that satiety can be attenuated simply by disrupting memory encoding during a meal. Consistent with this idea, eating in the presence of A distractor (for example, a computer game) affects not only appetite at the end of a meal^34,35^ but it also increases the amount of food that is consumed at a subsequent meal.^36,37^ Thus, it would appear that meal planning serves two roles. In the moment, it determines the portion size that is selected and then subsequently consumed. However, it also establishes a memory for portion size, which then affects the satiety that is experienced after a meal has been consumed. To isolate and quantify this second role, my collaborators and I manipulated the amount of soup that people consumed relative to the volume that they remembered consuming.^38^ Independent manipulation of ‘actual' and ‘perceived' volumes was achieved using a peristaltic pump connected covertly to a soup bowl. Before lunch, participants were shown either 300 ml or 500 ml of soup. Orthogonal to this, they consumed either 300 ml or 500 ml (yielding four conditions). Two and three hours after eating, differences in rated hunger reflected the amount of soup that participants remembered consuming (perceived amounts) rather than the actual amounts consumed, thereby exposing the important causal role of memory processes in satiety.

From the above, it also follows that the satiety response from a food might be enhanced simply by changing memory based on visual information about its formulation, function, energy content and so on. Accordingly, various forms of information and labelling are found to have a marked effect on subsequent hunger and fullness.^39, 40, 41^ In future, studies should consider the extent to which these sizable effects are preserved even after repeated exposure to a specific food or product.

## Expected satiety, palatability and self-selected food portions

Efficient meal planning also reduces waste. However, it also requires a capacity to be able to predict the consequences of consuming different food portions and to select an ‘ideal.' To understand this process, it is helpful to be able to measure expectations associated with the consumption of different foods. Early approaches relied on rating scales.^42,43^ More recently, techniques have been developed that quantify expectations very precisely by comparing foods directly on a calorie-for-calorie basis. The first of these uses a classical psychophysical approach based on a ‘method of constant stimuli'.^44^ Participants are shown a fixed ‘standard' portion of food and this is compared against a different ‘comparison' food. Over a series of trials the size of the comparison food is manipulated and participants are asked to pick the food that is expected to deliver greater satiety. At the end of the task a measure of ‘expected satiety' is calculated. This relates to the number of calories of the comparison food that would be expected to deliver the same satiety as the fixed standard. A conceptually similar alternative is to use a ‘method of adjustment'.^45^ Participants are shown a picture of a standard and a comparison food. Using specialist software, participants change the size of the comparison portion using a keyboard. Pictures are loaded with sufficient speed that the change in the comparison becomes ‘animated.' Participants are told to match the comparison food until both are expected to deliver the same satiety. A related concept is ‘expected satiation'—the extent to which two different foods are expected to deliver fullness at the end of a meal, again, when compared on a calorie-for-calorie basis. Expected satiation and expected satiety tend to be highly correlated.

In an initial study my colleagues and I compared the expected satiety of 18 different foods.^44^ The results were surprising. Some foods were expected to deliver five to six times more satiety than others. Foods that were less energy dense (for example, potatoes) were expected to deliver significantly more satiety than high energy-dense foods. To an extent, this difference reflects the range of energy densities that were compared (for example, chocolate confectionary vs potatoes). However, even when comparing only main-meal entrees, reliable differences were observed (for example, a nominal 200-kcal portion of pasta was expected to confer the same satiety as a 385-kcal portion of pizza). Participants often report that they find these responses very easy, perhaps reflecting the fact that they are making judgments that are highly practiced and rehearsed. We also see fine discrimination between foods, even in those that are very similar. For example, subtle changes to the viscosity of a yogurt drink generate reliable changes in expected satiety.^46^ Similarly, people clearly discriminate between different types of desserts^47^ and soups ^48^ based on their expected satiation.

The discovery that people are able to provide these precise estimates probably reflects the fact that they serve a purpose. Using a method of adjustment, it is also possible to obtain a measure of a person's momentary ideal portion size. Participants are shown a single image of a food and they manipulate its size until an ideal portion is shown. Ideal portion sizes are highly correlated with expected satiety and expected satiation,^45, 49^ suggesting that portions-size selection is governed primarily by these anticipated post-ingestive consequences.

Palatability is often mooted as an important determinant of food intake, overconsumption and weight gain.^50, 51, 52^ However, the relative role of palatability is rarely compared against other non-affective expectations and beliefs. In another study my colleagues and I explored the extent to which natural variation in the palatability of commonly consumed foods accounts for variance in the energy content of self-selected portion sizes.^45^ Medium- and high-energy-dense foods were compared. When assessed in this way, we found that variation in palatability explained a trivial proportion. By contrast, expected satiation was an especially good predictor (*r*=−0.8). One explanation is that the role of palatability has been overstated. It is exposed when highly palatable and highly unpalatable versions of the same food are compared. However, commonly consumed foods are rarely unpalatable. Therefore, the effect of a modest range in palatability may be swamped by a large range of variation in expected satiation.

A potential criticism is that measures of expected satiation and satiety are often taken using software and two-dimensional images. To address this concern measures derived from screen-based psychophysics have recently been assessed as predictors of actual food intake.^53^ Screen-based measures of expected satiety were highly correlated with subsequent self-selected portions of real food. Most people ate this self-selected portion in its entirety (90%) and, by contrast, measures of palatability were poor predictors of food intake.

## A role for learning

The fact that foods differ considerably in their expected satiety and expected satiation tells us something important. Expectations are not based solely on the energy content of food. Were this true, then all foods would have the same expected satiety and satiation. Instead, as already noted, low-energy-dense foods tend to have higher expected satiety and satiation. The macronutrient content of food may also be important. However, this remains to be established.^44^ In food-intake studies, energy intake is often reduced when participants are offered access to low-energy-dense foods.^54, 55, 56^ In part, this may be because people tend to consume the same volume of food irrespective of its energy content.^54^ One possibility is that expected satiety and expected satiation reflect a similar process—responses are governed by the perceived volume of a food which, in turn, is governed largely by its energy density. In a recent study, a variance partitioning approach revealed that the perceived volume of a food accounts for a large proportion of the relationship between expected satiation and self-selected portion sizes.^57^ However, a slightly larger proportion can also be considered unique and independent of the perceived physical dimensions of a food. One possibility is that expectations are governed by other related cues. For example, it would appear that the weight of a food independently influences its expected satiety and expected satiation.^58^ Other intriguing studies indicate that expected satiation reflects a complex relationship between ‘eating topography' (bite size, bite rate, swallow rate and so on) and actual satiation.^59^ High levels of expected satiation are associated with smaller bite sizes and with greater chewing.^60^

The fact that decisions about portion size are not governed simply by the physical dimensions of a food implies that judgments are learned. Both humans and non-human animals acquire flavour preferences based on an association that forms between the sensory characteristics of a food and its post-ingestive consequences.^61,62^ If a food provides strong nutrient reinforcement then this process generates a preference for its flavour. One possibility is that a similar process is responsible for moderating expected satiety and expected satiation. In a recent study, participants consumed a single portion of an otherwise identical high- or a low-energy-dense novel food.^63^ At a subsequent test session, expected satiation increased, but only in participants who had previously consumed the high-energy-dense version during training. This finding is exciting, because it suggests that flavour-nutrient associations are expressed in decisions about portion size prior to meal onset. In animals, similar associations are thought to influence the termination of a meal.^64^ Again, this distinction reinforces the notion that humans differ from other species because our meal size tends to be determined before meal onset rather than via feedback that is generated as a meal progresses.

Recently, it has also become clear that expectations drift over time. Across a range of studies, it would appear that familiarity increases the expected satiety and expected satiation of a food. This pattern is evident in single foods such as sushi,^65^ across a range of foods,^44^ and in children.^66^ Further, the degree of drift appears to be especially dramatic if a food has been eaten to fullness.^67^ This evidence for ‘expected-satiation drift'^65^ is potentially good news for the development of low-energy-dense products that are designed to reduce energy intake. Once expected satiety is learned, it tends to remain stable, even after repeated exposure to a low-energy-dense alternative.^68^ A potential drawback, however, is that any reduction in energy content will still be detected and, over time, this may reduce the reward value and hedonic quality of a food. This hypothesis is consistent with results from a recent study in humans^68^ and with the broader concept of the ‘missing calories effect,' which is observed in rodents.^69,70^ In future, researchers might consider the extent to which this process has a negative effect on the acceptability of commercially manufactured foods that are designed to confer benefits for weight loss.

## Concluding remarks

In this brief review, I have argued that the control of meal size is governed largely by a period of cognitive activity (planning) that occurs before a meal begins. I started by noting the importance of the portion-size effect, but also our uncertainty about the underlying mechanism. Based on the above discussion, I suggest that greater attention should be paid to the effects of large portions on the process of meal planning.

Taking this idea further, it may be worth considering whether other well-established phenomena are also reflected in decisions about portion size. One such example is the so-called ‘variety effect'.^71^ Consistently, studies show that food intake increases when there is more variety in a meal, perhaps because variety arrests the development of sensory specific satiety as the meal progresses.^72^ The same effect of variety is also observed in meal planning.^73^ Larger portions tend to be selected when variety is increased in a meal, either by introducing different types of food or by increasing the sensory contrast between its components. Again, this demonstrates a role for anticipatory control (planning) and it extends our characterisation of a well established phenomenon beyond a passive within-meal process.

We have also seen that portion-size decisions are complex, they are sophisticated, and they are governed by prior experience. Simply because they ‘feel' automatic and outside our control should not be taken to imply that control is absent. Indeed, the converse may be the case. One of the characteristics of highly practiced and rehearsed decisions is that they are made in the absence of conscious effort. *Prima facie,* it would appear that the portion-size effect is evidence for a human frailty—a tendency to be influenced by our environment, perhaps outside our own volition. This characterisation may be unhelpful because it oversimplifies a complex psychological process.
